# Time-Course Transcriptome Profiling of a Poxvirus Using Long-Read Full-Length Assay

**DOI:** 10.3390/pathogens10080919

**Published:** 2021-07-21

**Authors:** Dóra Tombácz, István Prazsák, Gábor Torma, Zsolt Csabai, Zsolt Balázs, Norbert Moldován, Béla Dénes, Michael Snyder, Zsolt Boldogkői

**Affiliations:** 1 Department of Medical Biology, Faculty of Medicine, University of Szeged, 6720 Szeged, Hungary; tombacz.dora@med.u-szeged.hu (D.T.); prazsak.istvan@med.u-szeged.hu (I.P.); torma.gabor@med.u-szeged.hu (G.T.); csabai.zsolt@med.u-szeged.hu (Z.C.); zsolt.balazs@uzh.ch (Z.B.); n.moldovan@amsterdamumc.nl (N.M.); 2 Department of Genetics, School of Medicine, Stanford University, Stanford, CA 94304, USA; mpsnyder@stanford.edu; 3 Veterinary Diagnostic Directorate, National Food Chain Safety Office, 1143 Budapest, Hungary; denesb@nebih.gov.hu

**Keywords:** vaccinia virus, long-read sequencing, nanopore sequencing, transcriptome profiling, gene expression

## Abstract

Viral transcriptomes that are determined using first- and second-generation sequencing techniques are incomplete. Due to the short read length, these methods are inefficient or fail to distinguish between transcript isoforms, polycistronic RNAs, and transcriptional overlaps and readthroughs. Additionally, these approaches are insensitive for the identification of splice and transcriptional start sites (TSSs) and, in most cases, transcriptional end sites (TESs), especially in transcript isoforms with varying transcript ends, and in multi-spliced transcripts. Long-read sequencing is able to read full-length nucleic acids and can therefore be used to assemble complete transcriptome atlases. Although vaccinia virus (VACV) does not produce spliced RNAs, its transcriptome has a high diversity of TSSs and TESs, and a high degree of polycistronism that leads to enormous complexity. We applied single-molecule, real-time, and nanopore-based sequencing methods to investigate the time-lapse transcriptome patterns of VACV gene expression.

## 1. Introduction

Poxviruses infect a wide variety of vertebrate and invertebrate species [1]. Among these, variola virus is the causative agent of smallpox [2] which was declared eradicated in 1980 by a vaccination program using the closely related vaccinia virus (VACV) as a live vaccine [3]. The variola genome exhibits roughly 90% sequence homology with VACV, which is the prototypic member of poxviruses. The VACV contains a 195 kbp double-stranded DNA molecule encoding more than 200 protein-coding genes. The viral genome is able to code for transcription factors and enzymes for DNA and RNA syntheses, and for capping [4] and polyadenylation [5] of RNAs, which allows VACV to replicate in the cytoplasm.

VACV genes can be classified according to their expression kinetics as early (E), intermediate (I), and late (L) genes [6,7,8,9]. A unique feature of poxviruses is that the entire transcription apparatus is packed into the virion, and thus E genes are expressed instantly following the entry to the host cells [10]. The E genes encode proteins needed for the synthesis of nucleic acid molecules, and also play roles in virus–host interactions. The next step is the onset of DNA replication in conjunction with the expression of first the I and then the L kinetic classes of genes. Transcription of I genes require de novo synthesis of E proteins, whereas L gene transcription is dependent on the expression of particular E and/or I genes. The post-replicative (PR) viral genes, including I and L genes, primarily specify the structural elements of VACV [11]. At the final stage of viral infection, the assembled and enveloped nucleocapsids egress from the host cells.

VACV genes were earlier classified on the basis of preference for co-localization on the viral DNA [12]. Particularly, E genes are clustered near the genomic termini [13], whereas the I and L genes are gathered at the central part of the viral DNA. Adjacent genes are mainly positioned at the same orientations; therefore, the number of convergent and divergent gene pairs is relatively low compared to, e.g., herpesviruses [14]. Assarsson and co-workers, using genome tiling arrays, showed that 35 VACV genes are expressed during the immediate-early (IE) stage of viral replication [12] and do not require de novo protein synthesis; therefore, the E genes should belong to the IE kinetic class [15], as in, e.g., herpesviruses [16] and baculoviruses [17]. However, this terminology, based on the distinction of IE class of genes, has not become widely accepted. Yang and co-workers subdivided E genes into E1.1 and E1.2 classes [18] based on cluster analysis, and suggested that the kinetic differences could be due to the relative affinity of transcription factors for promoters. Yang and colleagues [18] demonstrated that all early RNAs can be synthesized in the absence of de novo protein synthesis and therefore all early RNAs, not just the 35 listed by Assarson, can be termed IE. In addition to genome tiling array [19], VACV transcriptomes have also been studied using RNA-Seq [18], ribosome profiling (RP) [11,20] and microchip analyses [21,22]. In two consecutive studies, Yang and co-workers detected 118 E, 53 I and 38 L genes using short-read sequencing (SRS) [13,14]. RP analysis has been used for identifying translational initiation sites (TISs). A recent study on VACV transcriptome demonstrated that many TISs occur within the open reading frames (ORFs), in the 5′-untranslated regions (UTRs) and in the intergenic regions [11]. Nonetheless, these ORFs were short; therefore, the authors raised doubts about their biological significance. The above-mentioned techniques are unable to read full-length transcripts, and hence fail to provide a comprehensive description of transcriptomes. Even though the SRS approach produces satisfactory sequencing depth and coverage, the assemblies of RNAs are incomplete. The major difficulty in the characterization of the poxvirus transcriptome is to profile the extremely complex meshwork of transcriptional overlaps, including readthroughs and premature termination of RNA molecules, as well as the large variation in the transcription start sites (TSSs) and the transcriptional end sites (TESs). Hereafter, conventional sequencing techniques that are inefficient in detecting full-length transcripts considerably underestimate the complexity of the viral transcriptome.

Currently, two long-read sequencing (LRS) techniques—the Oxford Nanopore Technologies (ONT) and the Pacific Biosciences (PacBio)—rule the market. The special utility of LRS techniques is based on their ability to detect individual RNA molecules, which allows the efficient identification of long multi-ORF RNA molecules, alternatively transcribed and processed transcripts, and transcriptional overlaps. Despite this fact, annotation of transcript ends is still particularly challenging due to the vast complexity of the vaccinia virus transcriptome. The amplification-based Single-Molecule Real-time (SMRT) Iso-Seq technique applies a switching mechanism at the 5’ end of the RNA template, which allows the generation of full-length cDNAs [23]. A beneficial characteristic of the PacBio approach is that the errors are simply corrected due to the high consensus precision of this technique [24]. The advantages of the ONT sequencing approach over the PacBio technique include the cost-effectiveness, higher read output, and the capacity to read sequences within the range of 200 to 800 bps, in which both the SRS and PacBio approaches are less effective [25]. The major drawback of the ONT technique is the relatively high error rate, which is not necessarily disadvantageous to transcriptome analyses of well-annotated genomes, such as that of vaccinia virus [26]. Nonetheless, PacBio has much better sequencing accuracy than ONT. Thus, the combined use of these LRS methods eliminates the shortcomings of both, and they also serve as independent techniques for transcript validation. Our research group applied the ONT and PacBio LRS platforms to investigate the transcriptomes of herpesviruses, including pseudorabies virus [27,28], herpes simplex virus type 1 [29,30], varicella zoster virus [31], and human cytomegalovirus [32,33], as well as a baculovirus [34], a circovirus [35], a retrovirus [36], African swine fever virus [37], and the VACV [38]. These studies identified several novel transcripts and transcripts isoforms, multigenic RNA molecules, and transcriptional overlaps. Additionally, the utility of LRS for kinetic characterization of the viral transcriptome has been demonstrated [39,40].

In this study, we report the sequencing analyses of the VACV dynamic transcriptome using two LRS platforms, the ONT MinION and PacBio Sequel technologies.

## 2. Results

### 2.1. Long-Read Sequencing of Vaccinia Virus Transcriptome

**Figure 1:**
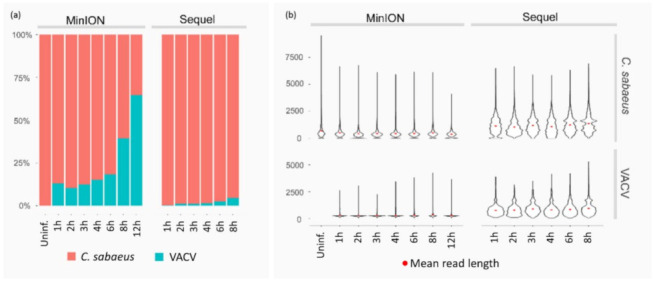
Read counts and lengths of uninfected and infected samples at each time point. (**a**) Fractions of reads mapped to host (C. sabaeus) and VACV genomes; dramatically reduced read counts of the host and an increased read count of the virus are observable with viral life cycle progression in MinION data, whereas similar but much smaller changes were detected in the Sequel dataset. (**b**) Mapped read lengths of the host and virus from MinION and Sequel sequencing from the uninfected sample and post-infection (p.i.) samples; the panel shows the effect of library preparation for PacBio sequencing, yielding longer reads with greater abundance than those from MinION sequencing.

In this work, the time-varying transcriptome of VACV was profiled using the PacBio isoform sequencing (Iso-Seq) template preparation protocol for Sequel platform, and the ONT sequencing 1D-Seq library preparation protocol for MinION platform. Both techniques are based on the amplification by polymerase chain reaction (PCR) prior to sequencing. For basecalling, we used the Albacore software developed by ONT. We also ran the Guppy sequencing pipeline (developed by ONT) for this purpose. These programs are based on the translation of electrical signals generated by the DNA strand passing through the nanopore into nucleotides. The difference between the two pieces of software is that the Guppy uses the graphic processing unit (GPU) in addition to the central processing unit (CPU), which results in faster basecalling but yields similar results [41]. We also compared the obtained data and found an almost perfect match (99.32% of TSSs, and 98.62% of TES positions of the Albacore-annotated viral transcripts were reobtained using the Guppy basecaller) between the results generated by the two basecallers. The obtained datasets were used to determine the coordinates of TSSs and TESs of VACV transcripts [42] applying the LoRTIA pipeline (https://github.com/zsolt-balazs/LoRTIA, accessed on 20 August 2019) that was developed by our research group [43]. Herein, we used a workflow and pipeline for transcriptome profiling of LRS datasets that were also developed in our laboratory [30,32,44]. The PacBio MagBead loading protocol selects DNA fragments of less than 1 kb [45]; therefore, the short monocistronic transcripts are underrepresented, or in some cases missing, in this dataset. PacBio Sequel and ONT MinION runs altogether resulted in 1,673,381 reads mapped to the virus (269,276) or to the host genome (1,404,105). Sequel sequencing analysis yielded 479,179 ROIs, of which 439,330 mapped to the host genome with an average mapped read length of 1,368 nt. The library preparation steps that mitigate loading bias of PacBio sequencing worked well for sample capture in host cells, but not so well for shorter VACV transcripts (Figure S1). In contrast, MinION sequencing had no preference for read length, resulting in a higher yield of short reads mapped to the virus and a more realistic change in host/virus read proportions at each p.i. time point (Figure 1). Dynamic gene expression profiles were classified by transforming normalized gene counts to a relative scale where the highest expression time point had a value of 1.0 (100%). Clustering was carried out using the k-means algorithm of the basic statistics package of R, version 3.5.

### 2.2. Dynamic VACV Transcriptome

#### 2.2.1. TSS and TES Dynamics

**Figure 2:**
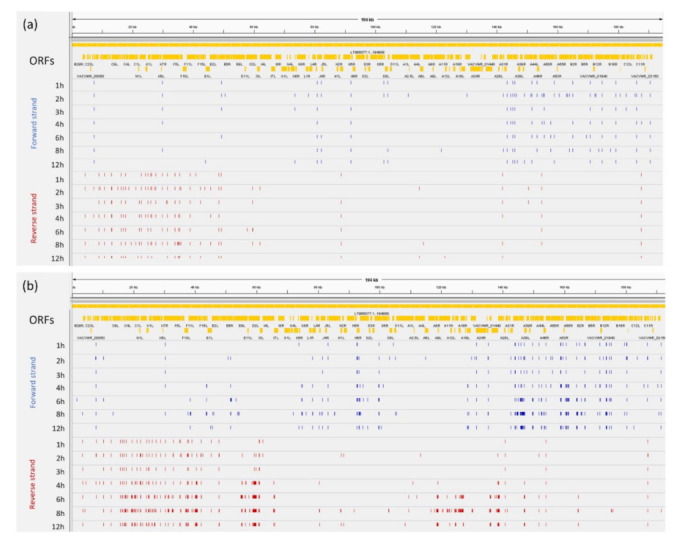
Genome-wide dynamics of VACV TSSs and TESs. (**a**) Blue dashes represent TSSs on the forward strand, while red dashes represent TSSs on the reverse strand. (**b**) Blue dashes represent TESs on the forward strand, while red dashes represent TESs on the reverse strand. (**a**,**b**) Yellow rectangles represent the ORFs.

In this part of our work, we analyzed the temporal changes in the use of transcription start and end sites of the viral RNA molecules annotated by the LoRTIA toolkit. The distribution of TSSs and TESs along the VACV genome is illustrated in Figure 2, which shows that TESs are expressed in larger diversity than TSSs, which is not the case, for example, in herpesviruses.

#### 2.2.2. Transcripts Kinetics

**Figure 3:**
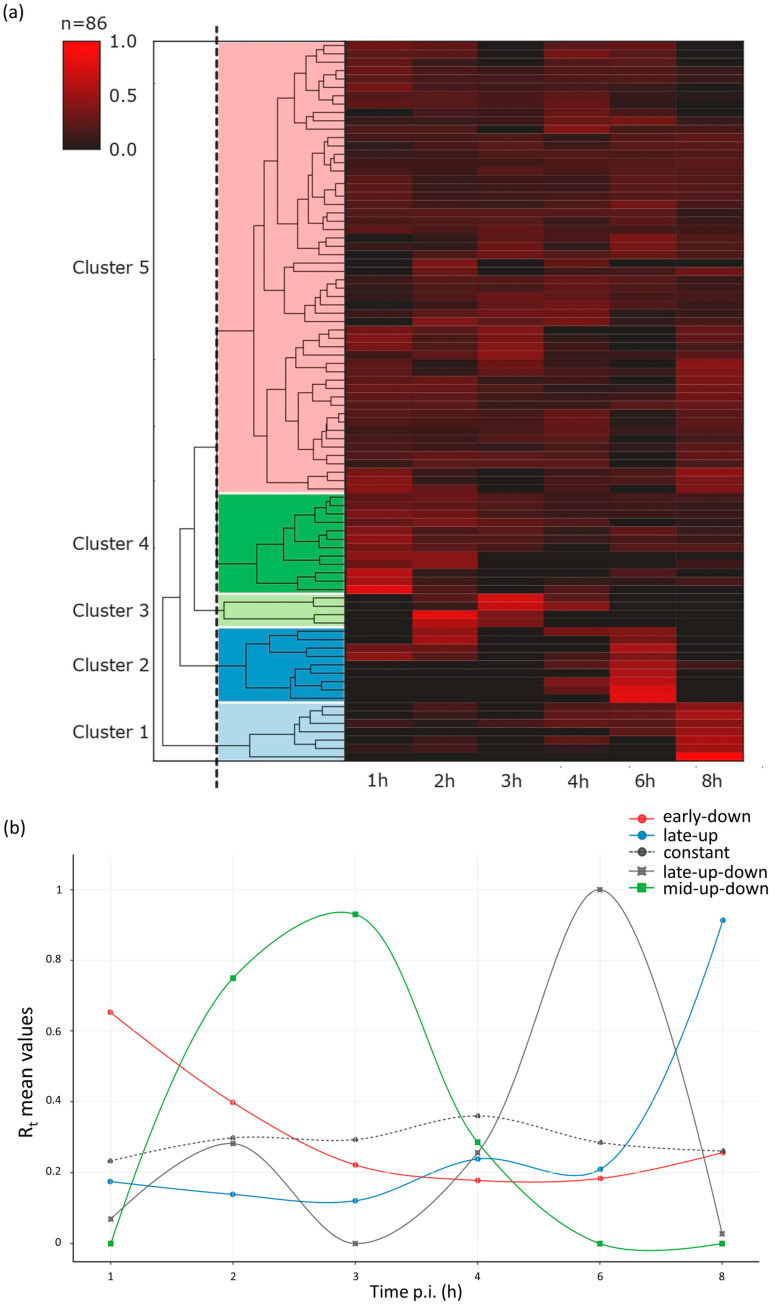
Expression profiles of the most abundant dynamic VACV transcripts and transcript isoforms. Rt values of the viral transcripts show distinct expression profiles in the five kinetic clusters. (**a**) The expression pattern of the viral transcript clusters. Five distinct VACV transcript clusters represented by a heatmap matrix. Each row represents changes in relative expression levels of an RNA. Red rectangles indicate high relative expression values and black rectangles indicate low relative expression values. (**b**) K-means clustering analysis was used to characterize the temporal expression of the five clusters obtained by hierarchical clustering. Relative Rt mean values of clusters are delineated in different time points of infection. All transcripts were identified using the LoRTIA pipeline. We note here that full-length sequencing provides a unique technology that is able to detect individual transcripts. Techniques lacking this ability (qPCR, short-read sequencing) cannot be used for the validation of the obtained results due to the high complexity of transcriptional overlaps in vaccinia virus.

**Figure 4:**
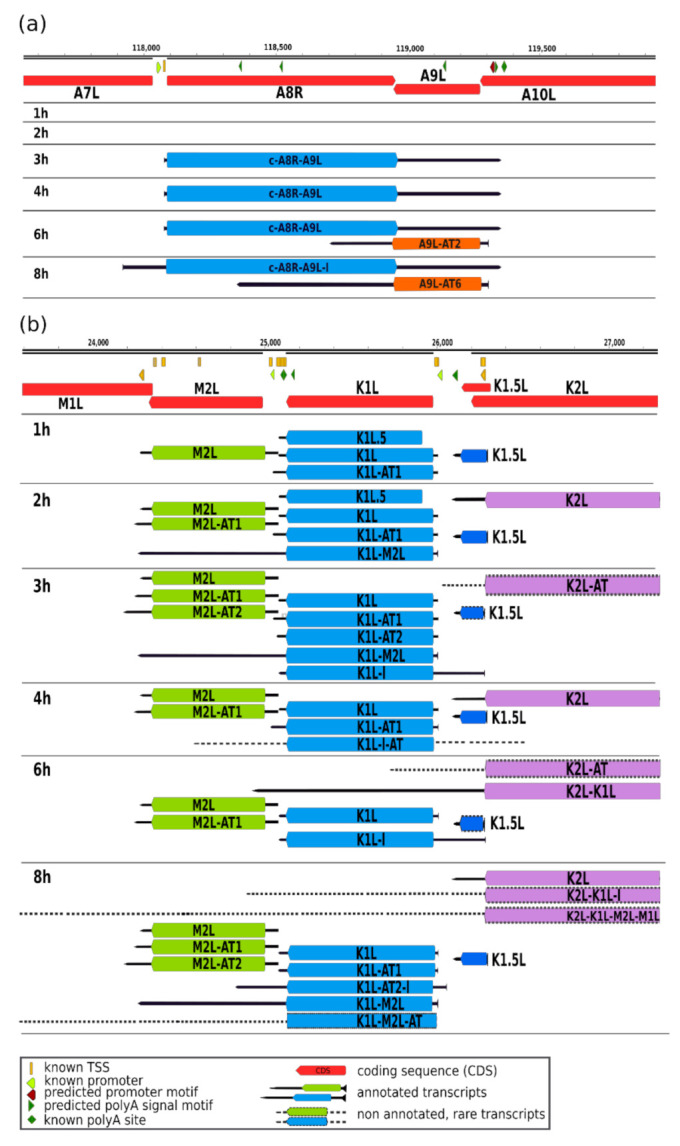
Dynamic VACV transcriptome. (**a**) Time-dependent changes of TSS isoforms of the A8R–A9L complex transcript and the most abundant TES isoforms of the A9L gene. The earliest time point when the LoRTIA pipeline identified the c-A8R–A9L complex RNA, which spans two oppositely oriented genes at this genomic region, was 3 h p.i. This transcript was also detected at 4 and 6 h p.i., but disappeared later. The A9L shorter 3′-UTR isoform was expressed at 6 h p.i., while its longer variant was detected at 8 h p.i.; (**b**) Expression levels of various transcripts and transcript isoforms within the M1L–K2L region, including transcriptional end site (TES) isoforms, mono- and bicistronic variants, and novel putative protein-coding genes. The characteristic feature of genes located in this genomic locus is that they produce longer 3′-UTR isoforms at later time points of infection; transcript structures and counts were determined using the LoRTIA software suite. See Table 1 for terminology.

**Figure 5:**
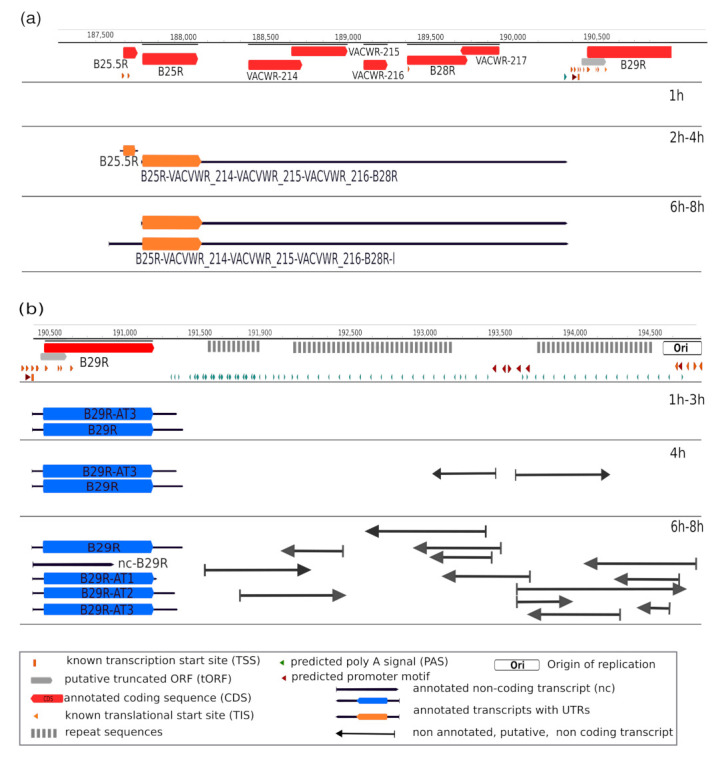
Expression dynamics VACV transcripts. (**a**) Kinetics of mono-, bi-, and polycistronic transcripts. The B25.5R–B29R genomic region contains eight ORFs of which no LoRTIA transcripts were detected at 1 h p.i. A monocistronic (B25.5R) and a pentacistronic transcript (B25R–B28R) were expressed at 2–4 h p.i. interval. At later time points, the pentacistronic RNA and its longer overlapping partner ((**a**), hexacistronic transcript) are expressed; however, the monocistronic transcript disappeared. (**b**) Expression dynamics of the replication-associated RNAs. Several transcripts were detected at the two ends of the VACV genome, around the replication origin of the virus. These transcripts appeared at 4 h p.i., following the initiation of the DNA replication, which suggests that they might have a regulatory role in this process. See Table 1 for terminology.

As a major advantage, LRS methods perform end-to-end sequencing of entire RNA or cDNA molecules, allowing identification and kinetic characterization of transcript isoforms. We performed kinetic analyses of LoRTIA transcripts (transcripts identified with LoRTIA) using k-means and hierarchical clustering and identified five distinct clusters in the dataset (Figure 3a). K-means clustering was used to describe the temporal expression levels of these clusters (Figure 3b), and the following expression profiles were obtained: (1) the ‘early-down’ group had continuously decreasing Rt values (see Methods) from the start of infection; (2) the ‘mid-up-down’ cluster had a local maximum between 2 and 3 h p.i.; (3) the ‘late-up-down’ cluster of transcripts reached maximum of Rt values at 6 h p.i.; (4) the ‘late-up’ cluster of transcripts had a clear late activity reaching maximum Rt values at 8 h p.i., and (5) most viral transcripts exhibited minor changes throughout the viral life cycle (‘constant’ cluster) (Table S1). The dynamics of various transcript categories are presented in Figure 4 and Figure 5, which show TSS and TES isoforms, polycistronic RNAs, and Ori-associated transcripts. Many RNA isoforms and mono- and polycistronic transcripts are differentially expressed throughout the viral life cycle.

#### 2.2.3. Dynamic Expression of ORFs

**Figure 6:**
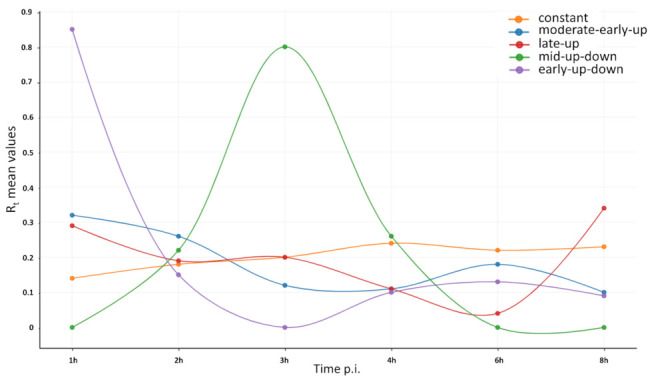
Categories of the expression curves of the vaccinia virus transcripts containing the same ORFs. Based on their dynamics, VACV genes can be grouped into five distinct clusters. The limitation of this approach is that only the abundant LoRTIA transcripts can be used for the analysis, while those lower-abundance overlapping transcripts that are not identified by the LoRTIA software are missing from the calculation. Other techniques (e.g., short-read sequencing, real-time RT-PCR) are not suitable for distinguishing between the overlapping transcripts and transcript isoforms; therefore, data generated by these techniques are not comparable with our results.

We summed transcripts with given ORFs alone or in the most upstream position of a polycistronic transcript (downstream ORFs were untranslated) and showed similar ORF kinetics to those of their canonical transcript isoforms. Hence, the relative proportions of other transcript isoforms were generally low relative to the main isoforms, and therefore had no significant effects on the expression dynamics of ORFs. We identified 57 VACV ORF using the strict criteria (detection of at least five LoRTIA transcripts). Applying K-means clustering, we clustered the ORFs into five clusters according to normalized gene expression profiles (Figure 6, Table S2). The five distinct gene expression clusters are as follows: (1) ‘early-up-down’ genes express a very high level at an early stage of infection; (2) ‘moderate-early-up’ genes have their maximum at an early stage of infection, but the difference between the expression levels at 1 h p.i. and the next time point is less than in the ‘early-up-down’ group; (3) genes belonging to the ‘mid-up-down’ category reach their maximum Rt value at 3 h p.i.; (4) members of ‘late-up’ gene cluster have their maximum Rt values at 6 h p.i.; and (5) the genes within the ‘constant’ cluster show relatively stable expression values. The LoRTIA software identified only a small fraction of VACV transcript isoforms, especially at the ‘chaotic region’ of the viral genome (this is the middle genomic region containing I and L genes, which produces extremely high transcript end diversity). Transcripts with extremely polymorphic TSS and TES were, among others, as follows: O1L, O2L, A10L, A11R, A12L, and A18R [42]. All LoRTIA-identified transcripts were encoded by genes previously categorized as E genes by Yang and colleagues [13]. The only exception was the A29L gene. The reason for the enrichment of the E genes in this LoRTIA dataset is that E genes produce transcripts with relatively exact TSSs and TESs, and therefore they were identified by the LoRTIA suit. Most ORFs that met the LoRTIA criteria produced transcripts from 1 h p.i. (exept A49R, C12L, VACWR_161, VACWR_183, and E2.5L). Some genes have not been categorized previously (A43R.5, C1.7L,,H3L.5,,E5.7R, E2.5L, E4.7L).

#### 2.2.4. Dynamics of the Transcriptional Readthroughs

TES isoforms are produced by the passing of at least one transcription termination signal by the RNA polymerase, yet the temporal expression of TES variants differed between genes (Figure S2). For some genes, all transcript isoforms appeared at every time point (WACVR_15, H5R, and their isoforms). In other genes, certain isoforms were expressed at only a single time point (N1L and B19R) or at multiple time points (WACVR_15 and H5R). According to our strict criteria, the A37R gene exhibited the highest variation in 3′-UTR with seven alternative ends. With some exceptions (e.g., WACVR_15), the expression profiles of 3′-UTR variants differed from each other throughout the viral life cycle.

We observed that the early genes, generally located in the ‘regular’ genomic regions (genome ends), exhibited a much lower level of TES polymorphism (in other words, transcriptional readthrough events) than late genes located at the ‘chaotic’ region (genome center). However, this phenomenon is underestimated in our analysis because, although there is a high read coverage at the ‘chaotic’ region, individual transcripts are difficult to identify due to the high transcript end diversity.

## 3. Discussion

Short-read sequencing has become a prevailing technique for transcriptome profiling [46,47]. However, comprehensive annotation from transcript datasets using this approach remains challenging [48]. LRS techniques are able to determine full-length transcripts in single reads without computational inference, and therefore easily identify multi-ORF RNAs, transcript isoforms (splice and length variants), transcript overlaps, and multigenic transcripts [49].

In this work, PacBio and ONT sequencing platforms were employed for the analysis of the transcription kinetics of VACV. Long-read RNA sequencing data were annotated by the LoRTIA pipeline, developed in our laboratory. In order to avoid annotation of spurious reads as transcripts, we further filtered the obtained data using very strict criteria [42]. We assume that a large fraction of excluded reads may represent existing transcripts. In contrast to herpesviruses [29,31], but similarly to baculoviruses [34], in vaccinia virus the heterogeneity of TESs exceeds that of TSSs. The overall polymorphism of length isoforms and the complexity of transcriptional overlaps are also higher in VACV than in other viruses. The high occurrence of within-ORF TSSs is also unique to VACV. We obtained that the canonical transcripts are overrepresented among the isoforms encoded in the same genomic loci of VACV, which raises the question as to whether these isoforms might be mere transcriptional noise without any function which are produced by cryptic promoters or by error-prone transcriptional apparatus. However, the number of transcripts containing the same ORFs but different TSSs and/or TESs is likely to be significantly underestimated because a large number of low-abundance RNA molecules are unidentified by the LoRTIA software, and therefore the ratio of non-canonical to canonical transcripts may be much higher than we detected. Furthermore, most RNA molecules comprise multiple ORFs, and most transcript isoforms contain unique combinations of canonical ORFs and upstream ORFs, suggesting that this transcriptional variety is functional.

Transcriptional overlaps and readthroughs have been proposed to play a role in genome-wide regulation of gene expression through the collision and/or competition between the transcriptional machineries [50]. Additionally, it has been shown in a yeast model that duplexes formed by the binding of overlapping 3′-UTRs of convergent transcripts play a role in the modulation of mRNA expression by limiting their access to translational repressors [51]. It is also possible that these RNA duplexes activate RNA interference and thereby regulate gene expression at the translation level [52]. It has been reported that, in contrast to the 5′-UTR region of the short TSS isoforms, the longer TSS variants contain upstream ORFs, which increases the coding potential of viral genes [32]. The longer 5′-UTR and 3′-UTR regions may also contain other regulatory sequences, which enable the transcripts isoforms harboring these extra sequencing to play a differential role in the viral pathogenesis. Further investigations are needed to evaluate the biological functions of transcript isoforms and the transcriptional readthrough.

Although many VACV transcripts are structurally polycistronic, they are presumably functionally monocistronic; that is, only the first ORF at the 5′ end is translated.

In this study, we analyzed the kinetic properties of TSSs and TESs of RNA molecules, the ORFs and the transcripts themselves. In general, the conventional kinetic categorization of viral genes is based on two considerations: (1) at what stage of the viral life cycle the given ORF exhibits a high expression level, (2) and the dependency of gene expression on newly synthesized viral transcription factors or on the DNA replication, which can be examined by adding inhibitors of translation or DNA polymerase, respectively. However, earlier categorization had severe limitations, which were based on the fact that viral transcripts exhibit extensive overlapping patterns. Short-read sequencing and qPCR are unable to distinguish between the co-oriented overlapping transcripts, while those techniques which are able to detect the individual transcripts (e.g., Northern blot analysis) have low precision and resolution. Our long-read approach is able to distinguish between overlapping transcripts and generate highly accurate data.

In this work, the abundant VACV transcripts were kinetically categorized according to their expression dynamics and they were grouped into five distinct clusters (‘early-up’, ‘late-up’, ‘constant’, ‘late-up-down’, and ‘mid-up-down’; Figure 3b). Viral ORFs were also categorized using the following method: read counts of abundant, LoRTIA-identified transcripts, which contain the same ORF, were summed and these were used for the calculation of the relative expression values at each time point. Based on the dynamic pattern of ORFs, using k-means clustering, we created five distinct groups (‘early-up-down’, ‘moderate-early-up’, ‘mid-up-down’, ‘late-up’, and ‘constant’; Figure 6). We note here, that—in contrast to the transcript kinetics—the ORF kinetics are inaccurate because a large fraction of transcript isoforms is unidentified by the LoRTIA suit; therefore, transcript coverages are significantly underestimated in the viral genes.

Our data show that many genes, located in the ‘regular’ genomic region and producing regular LoRTIA transcripts, have at least two TES isoforms (Figure S2). The longer transcript isoforms generate readthroughs into the downstream genes. Readthroughs are also produced at the ‘chaotic’ segment of the genome by the L genes; however, because of the large extent of transcript end polymorphism, they are not identified as a transcript by the LoRTIA software.

Collectively, in this study, we demonstrate the utility of the LRS approach for the kinetic analysis of viral transcripts. However, our results are difficult to compare with the conventional categories because, in contrast to these techniques, LRS is able to detect individual transcripts. Due to the complex TSS and TES combinations of individual VACV transcripts, a large fraction of them is undetected by the LoRTIA program. The large extent of the alternative use of transcript ends and in-frame ATGs has yet to be functionally elucidated.

## 4. Materials and Methods

### 4.1. Cells and Viruses

African green monkey (Chlorocebus sabaeus) kidney fibroblast cells (CV-1; obtained from the American Type Culture Collection) were used for the propagation of the Western Reserve strain of vaccinia virus (VACV). Cells were incubated at 37 °C in a humidified atmosphere containing 5% CO_2_ until confluency was reached (a density of 2 × 10^7^ cells per 150 cm^2^ tissue culture flask). Cells were cultured in RPMI 1640 medium supplemented with 10% fetal bovine serum and antibiotic–antimycotic solution (Sigma-Aldrich). Subsequently, cells were rinsed with serum-free RPMI medium prior to infection with the virus diluted in serum-free RPMI medium.

### 4.2. Infection

CV-1 cells were infected with 3 mL aliquots of VACV solution at a multiplicity of infection (MOI) of 10 pfu/cell, and were incubated at 37 °C in an atmosphere containing 5% CO_2_ for 1 h with brief agitations at 10 min intervals to redistribute virions. Subsequently, the infected cells were rinsed with PBS, which was followed by adding complete growth medium (RPMI + 10% FBS) to tissue culture flasks. The cells were then incubated at 37 °C for 1, 2, 3, 4, 6, 8, and 12 h in a humidified atmosphere containing 5% CO_2_. Following incubation, media were removed and the cells were rinsed with serum-free RPMI 1640 medium and were subjected to three cycles of freeze–thawing. Finally, cells were scraped into 2 mL aliquots of PBS and stored at −80 °C until use.

### 4.3. RNA Purification

Total RNA was extracted from VACV-infected cells at various stages of viral infection between 1–16 h p.i. using Macherey–Nagel RNA kits according to the manufacturer’s instructions. Polyadenylated fractions were isolated from total RNA samples using Oligotex mRNA Mini Kits (Qiagen, Hilden, Germany) following the Oligotex mRNA Spin-Column Protocol.

### 4.4. PacBio Sequel Sequencing

#### 4.4.1. cDNA Synthesis

Complementary DNA was generated from polyA(+) RNA fractions using SMARTer PCR cDNA Synthesis Kits (Clontech, Mountain View, CA, USA) according to PacBio Isoform Sequencing (Iso-Seq) using Clontech SMARTer PCR cDNA Synthesis Kit and No Size Selection protocols. The samples from 1, 2, 3, 4, 6, and 8 h p.i. were used individually to produce libraries for Sequel sequencing. The samples were used for SMRTbell template preparation using the PacBio DNA Template Prep Kit 1.0 (Menlo Park, CA, USA).

#### 4.4.2. Purification of Samples

Bead-based cDNA purification (Agencourt AMPure XP; Beckman Coulter) was applied after each enzymatic step.

#### 4.4.3. SMRTbell Library Preparation and Sequencing

The detailed version of the template preparation protocol is described in our earlier publication [53]. Briefly, primer annealing and polymerase-binding reactions were performed using Sequel Sequencing Kit 2.1 (PacBio, Menlo Park, CA, USA). and Sequel DNA Polymerase 2.0 (PacBio, Menlo Park, CA, USA). were applied for sequencing on the Sequel. Polymerase–template complexes were bound to MagBeads prior to loading into the PacBio instrument. Reactions were then performed using PacBio’s MagBead Kit (v2) (PacBio, Menlo Park, CA, USA). Finally, 600 min movies were captured using Sequel and one movie was recorded for each SMRTcell.

### 4.5. ONT MinION Sequencing

#### 4.5.1. D cDNA Library Preparation

PolyA(+) RNA fractions were used for cDNA sequencing on the ONT MinION device. RNAs from different infection time points were converted to cDNAs according to the ONT 1D strand-switching cDNA ligation protocol (Version: SSE_9011_v108_revS_18Oct2016). Libraries were generated using the above-mentioned 1D ligation kit and protocol, the Ligation Sequencing 1D kit (SQK-LSK108, Oxford Nanopore Technologies, Oxford, UK), and the NEBNext End repair/dA-tailing Module NEB Blunt/TA Ligase Master Mix (New England Biolabs, Ipswich, MA, USA) according to the manufacturer’s instructions. Briefly, polyA(+)-selected RNAs were converted to cDNAs using poly(T)-containing anchored primers ((VN)T20; Bio Basic, Markham, ON, Canada), dNTPs (10 mM, Thermo Scientific, Waltham, MA, USA), Superscript IV Reverse Transcriptase Kit (Life Technologies, Carlsbad, CA, USA), RNase OUT (Life Technologies, Carlsbad, CA, USA), and strand-switching oligonucleotides with three O-methyl-guanine RNA bases (PCR_Sw_mod_3G; Bio Basic, Markham, ON, Canada). The resulting double-stranded cDNAs were amplified by PCR using KAPA HiFi DNA Polymerase (Kapa Biosystems, Wilmington, MA, USA), Ligation Sequencing Kit Primer Mix (provided by the ONT 1D Kit, ONT, Oxford, USA), and a Veriti Thermal Cycler (Applied Biosystems, Waltham, MA, USA). The NEBNext End repair/dA-tailing Module (New England Biolabs, Ipswich, MA, USA) was used to blunt and phosphorylate cDNA ends, and the NEB Blunt/TA Ligase Master Mix (New England Biolabs, Ipswich, MA, USA) was used for adapter (supplied in the 1D kit) ligation.

Barcoding: Individually sequenced cDNAs were barcoded using a combination of the following ONT protocols: the 1D protocol was used until the first end-preparation step, then we switched to the 1D PCR barcoding (96) genomic DNA (SQK-LSK108) protocol (version: PBGE96_9015_v108_revS_18Oct2016, updated 25 October 2017), followed by the barcode ligation step using the ONT PCR Barcoding Kit 96 (EXP-PBC096) (ONT, Oxford, UK). Barcode adapters were ligated to end-prepped samples using Blunt/TA Ligase Master Mix (New England Biolabs).

#### 4.5.2. Purification of cDNAs

Samples were purified using Agencourt AMPure XP magnetic beads (Beckman Coulter) after enzyme reaction steps during library preparation.

#### 4.5.3. Sequencing on the MinION Device

ONT cDNA libraries were loaded onto 3 and 2 ONT R9.4 SpotON Flow Cells for sequencing, respectively. Sequencing runs were performed using MinKNOW.

### 4.6. Nucleic Acid Quality and Quantity Checking

Concentrations of the reverse-transcribed and adapter-ligated RNAs were measured using a Qubit 2.0 Fluorometer (Life Technologies, Carlsbad, CA, USA). as in [42]. An Agilent Bioanalyzer 2100 (Agilent Technologies, Santa Clara, CA, USA) was used for quality control of RNA samples.

### 4.7. Data Analysis and Visualization

#### 4.7.1. Generation of Consensus Sequences from the PacBio Dataset

ROI reads were created from the RSII raw data using the RS_ReadsOfInsert protocol (SMRT Analysis v2.3.0) with the following settings: Minimum Full Passes = 1, Minimum Predicted Accuracy = 90, Minimum Length of Reads of Insert = 1, Maximum Length of Reads of Insert = No Limit. ROIs from the Sequel dataset were generated using SMRT Link 5.0.1.9585.

#### 4.7.2. ONT Dataset—Basecalling

MinION basecalling was performed using the ONT Albacore software (Albacore v.2.0.1) (ONT, Oxford, UK). The ONT’s Guppy (Guppy v.3.6.0) (ONT, Oxford, UK was also used for basecalling with the aim to validate the annotated transcripts. Transcripts detected by both (Albacore and Guppy) datasets were used in this study.

#### 4.7.3. Mapping and Annotation of TSS and TES Positions and Transcripts

PacBio ROIs and ONT raw reads were aligned to the reference genome of the virus (LT966077.1) and to that of the host cell (Chlorocebus sabaeus; GenBank assembly accession: GCA_000 409795.2 (latest); RefSeq assembly accession: GCF_000 409795.2 (latest)) using minimap2 aligner (version 2.13) with the options -ax splice -Y -C5–cs. After applying the LoRTIA toolkit (https://github.com/zsolt-balazs/LoRTIA, accessed on 20 August 2019), the determined 5′ and 3′ ends of transcripts and detected full-length reads were mapped. For analyses of transcript dynamics, we included only transcripts that were validated by two different library preparation techniques (PacBio Iso-Seq and MinION 1D or PacBio Iso-Seq and Lexogen TeloPrime or MinION 1D and Lexogen TeloPrime).

### 4.8. Transcript Nomenclature

Novel transcripts and ORFs were named according to our previous publication on structural analysis of VACV transcriptome [42], using a scheme that incorporates existing ORF names, according to the common (Copenhagen) HindIII fragment letter/number-based nomenclature [54]. Previously described ORFs were not renamed and our scheme allowed for future additions of novel transcripts. Multicistronic transcripts were named for all contributed ORFs, and the most upstream ORF was listed first. Non-coding transcripts are identified with the prefix ‘nc’, and complex transcripts carry the prefix ‘c’, When a non-coding transcript was antisense to the ORF for which it was named, an ‘as’ prefix was added. Names of length isoforms, e.g., TSS and TES isoforms, end with ‘l’ for long, ‘s’ for short, or ‘AT’ for alternative termination (S1 Note). Isoform categories are presented in Table 1.

**Table 1:** Terminology of the VACV transcripts. X refers to any VACV gene.

Type of Transcript	Naming Scheme
given CDS	X1R
most abundant transcript isoform, probably the main RNA isoform of a given CDS	X1R
novel genes between two previously annotated genes	X1.5R
novel, embedded, potentially protein-coding gene	X1R.5
new RNA isoform with an alternative TES, resulting in shorter or longer 3’ UTR	X1R-AT
long 5’-UTR isoform	X1R-l
short 5’-UTR isoform	X1R-s
non-coding RNA isoform terminating before the stop codon of a known ORF or transcript without ORF	nc-X1R
3’-truncated transcript isoform within a CDS, without start codon	tr-X1R
complex transcript isoform, spanning oppositely oriented ORFs	c-X1R-X2L
antisense transcript isoform, oriented oppositely to the given ORF	as-X2R

### 4.9. Calculation of Relative Expression Levels of Viral Transcripts

Criteria were set to count relative viral expression values. Specifically, only transcript isoforms annotated by LoRTIA were used for the calculation. In further analyses, criteria were extended to include only transcripts that were represented by at least five independent full-length reads. Due to the high variation in their TSSs, the postreplicative genes at the ‘chaotic’ genomic region were underrepresented at the late stage of viral infection [42].

Normalized relative expression ratios at a given time point of infection (Rt) were calculated using the following formula, $Rt={\left(\frac{C}{\sum_{i=1}^{n}C}\right)}^{-h}$, where n = number of different transcript isoforms in a given sample, C = the read count of a given transcript at a given time point, and $h=\overset{k}{\underset{i=1}{\sum }}C\;$ returns the mean C value of a given transcript at time points *k* = 1, 2, 4, 6, or 8 h. Rt values were clustered hierarchically using the Instant Clue statistic program [55] to determine numbers of transcript isoform clusters. The Euclidian metric was used to cluster rows in the data frame and thereafter k-means hierarchical clustering was conducted with 100,000 repetitions. Means of each cluster were presented in BioVinci 1.1.5. Transcripts with at least five full-length reads were considered in cluster analyses. For functional analyses of transcript isoform coding of known VACV proteins, we used descriptions from the UniProt database (https://www.uniprot.org/, accessed on 20 October 2009).

### 4.10. Analysis of the Expression Dynamics of VACV ORFs

Normalized relative expression ratios (Rt -values) of ORFs at given time points of infection were calculated as described in the previous section with minor modifications. Briefly, Rt values of the ORFs were calculated by summing Rt values of transcript isoforms that harbor the same ORFs, with the exception of polycistronic transcripts carrying the given ORF in a downstream position. These transcripts were not translated.

## Data Availability

All data were uploaded to European Nucleotide Archive under the accession number PRJEB26434 (Characterization of the Vaccinia virus transcriptome) and PRJEB26430 (Dynamic characterization of the Vaccinia virus transcriptome).

## Supplementary Materials

The following are available online at https://www.mdpi.com/article/10.3390/pathogens10080919/s1, Figure S1: Mapped length distributions of MinION and Sequel sequencing reads. The upper halves of the density plots of VACV reads from MinION sequencing are not shown. MinION is well-suited for sequencing of smaller VACV RNAs, which are lost during library preparation for the Sequel platform, Figure S2: Dynamics of readthroughs. VACV genes and the associated 3′-UTR isoforms show diverse transcription profiles. This heatmap-like illustration shows the relative expression ratio of the various transcripts. White: no expression; light blue: low expression level; dark blue: maximum expression level of the given ORF; Table S1: VACV gene expression. (a) Relative expression values of the viral genes; the genes are arranged according to their kinetic clusters. (b) Detailed information about the function of examined VACV genes, Table S2: Gene expression kinetic profiles of VACV genes. Fifty-seven VACV genes (most of them have been categorized as E) were grouped into five distinct clusters according to their gene expression dynamics using k-means clustering.

## References

[1] Moss B., Knipe B.M., Howley P.M., Cohen J.I., Griffin D.E., Lamb R.A., Martin M.A., Rancaniello V.R., Roizman B. (2013). Poxviridae. Fields Virology.

[2] Esposito J.J., Sammons S.A., Frace A.M., Osborne J., Olsen-Rasmussen M., Zhang M., Govil D., Damon I.K., Kline R., Laker M. (2006). Genome Sequence Diversity and Clues to the Evolution of Variola (Smallpox) Virus. Science.

[3] Jacobs B.L., Langland J.O., Kibler K.V., Denzler K.L., White S.D., Holechek S.A., Wong S., Huynh T., Baskin C.R. (2009). Vaccinia virus vaccines: Past, present and future. Antivir. Res..

[4] Wei C.M., Moss B. (1975). Methylated nucleotides block 5-terminus of vaccinia virus mRNA. Proc. Natl. Acad. Sci. USA.

[5] Kates J., Beeson J. (1970). Ribonucleic acid synthesis in vaccinia virus: I. The mechanism of synthesis and release of RNA in vaccinia cores. J. Mol. Biol..

[6] Davison A.J., Moss B. (1989). Structure of vaccinia virus early promoters. J. Mol. Biol..

[7] Davison A.J., Moss B. (1989). Structure of vaccinia virus late promoters. J. Mol. Biol..

[8] Baldick C.J., Keck J.G., Moss B. (1992). Mutational analysis of the core, spacer, and initiator regions of vaccinia virus intermediate-class promoters. J. Virol..

[9] Broyles S.S. (2003). Vaccinia virus transcription. J. Gen. Virol..

[10] Moss B., Knipe D.M., Howley P.M. (2007). Poxviridae: The viruses and their replication. Fields Virology.

[11] Yang Z., Cao S., Martens C.A., Porcella S.F., Xie Z., Ma M., Shen B., Moss B. (2015). Deciphering Poxvirus Gene Expression by RNA Sequencing and Ribosome Profiling. J. Virol..

[12] Assarsson E., Greenbaum J.A., Sundström M., Schaffer L., Hammond J.A., Pasquetto V., Oseroff C., Hendrickson R.C., Lefkowitz E.J., Tscharke D.C. (2008). Kinetic analysis of a complete poxvirus transcriptome reveals an immediate-early class of genes. Proc. Natl. Acad. Sci. USA.

[13] Yang Z., Bruno D.P., Martens C.A., Porcella S.F., Moss B. (2010). Simultaneous high-resolution analysis of vaccinia virus and host cell transcriptomes by deep RNA sequencing. Proc. Natl. Acad. Sci. USA.

[14] Yang Z., Reynolds S.E., Martens C.A., Bruno D.P., Porcella S.F., Moss B. (2011). Expression Profiling of the Intermediate and Late Stages of Poxvirus Replication. J. Virol..

[15] Cooper J.A., Moss B. (1979). In vitro translation of immediate early, early, and late classes of RNA from vaccinia virus-infected cells. Virology.

[16] Honess R.W., Roizman B. (1975). Regulation of herpesvirus macromolecular synthesis: Sequential transition of polypeptide synthesis requires functional viral polypeptides. Proc. Natl. Acad. Sci. USA.

[17] Ross L., Guarino L.A. (1997). Cycloheximide Inhibition of Delayed Early Gene Expression in Baculovirus-Infected Cells. Virology.

[18] Yang Z., Bruno D.P., Martens C.A., Porcella S.F., Moss B. (2011). Genome-Wide Analysis of the 5′ and 3′ Ends of Vaccinia Virus Early mRNAs Delineates Regulatory Sequences of Annotated and Anomalous Transcripts. J. Virol..

[19] Rubins K.H., Hensley L.E., Bell G.W., Wang C., Lefkowitz E.J., Brown P.O., Relman D.A. (2008). Comparative analysis of viral gene expression programs during poxvirus infection: A transcriptional map of the vaccinia and monkey pox genomes. PLoS ONE.

[20] Stern-Ginossar N., Weisburd B., Michalski A., Le-Trilling V.T.K., Hein M., Huang S.-X., Ma M., Shen B., Qian S.-B., Hengel H. (2012). Decoding Human Cytomegalovirus. Science.

[21] Guerra S., Lopez-Fernandez L., Pascual-Montano A., Muñoz M., Harshman K., Esteban M. (2003). Cellular Gene Expression Survey of Vaccinia Virus Infection of Human HeLa Cells. J. Virol..

[22] Brum L.M., Lopez M., Varela J.-C., Baker H.V., Moyer R.W. (2003). Microarray analysis of A549 cells infected with rabbitpox virus (RPV): A comparison of wild-type RPV and RPV deleted for the host range gene, SPI-1. Virology.

[23] Zhu Y., Machleder E., Chenchik A., Li R., Siebert P. (2001). Reverse Transcriptase Template Switching: A SMART™ Approach for Full-Length cDNA Library Construction. Biotechniques.

[24] Miyamoto M., Motooka D., Gotoh K., Imai T., Yoshitake K., Goto N., Iida T., Yasunaga T., Horii T., Arakawa K. (2014). Performance comparison of second- and third-generation sequencers using a bacterial genome with two chromosomes. BMC Genom..

[25] Larkin J., Henley R.Y., Jadhav V., Korlach J., Wanunu M. (2017). Length-independent DNA packing into nanopore zero-mode waveguides for low-input DNA sequencing. Nat. Nanotechnol..

[26] Prazsák I., Tombácz D., Szűcs A., Dénes B., Snyder M., Boldogkői Z. (2018). Full Genome Sequence of the Western Reserve Strain of Vaccinia Virus Determined by Third-Generation Sequencing. Genome Announc..

[27] Tombácz D., Csabai Z., Oláh P., Balázs Z., Likó I., Zsigmond L., Sharon D., Snyder M., Boldogkői Z. (2016). Full-Length Isoform Sequencing Reveals Novel Transcripts and Substantial Transcriptional Overlaps in a Herpesvirus. PLoS ONE.

[28] Moldován N., Tombácz D., Szűcs A., Csabai Z., Snyder M., Boldogkői Z. (2018). Multi-Platform Sequencing Approach Reveals a Novel Transcriptome Profile in Pseudorabies Virus. Front. Microbiol..

[29] Tombácz D., Csabai Z., Szűcs A., Balázs Z., Moldován N., Sharon D., Snyder M., Boldogkői Z. (2017). Long-read isoform sequencing reveals a hidden complexity of the transcriptional landscape of Herpes simplex virus type 1. Front. Microbiol..

[30] Tombácz D., Moldován N., Balázs Z., Gulyás G., Csabai Z., Boldogkői M., Snyder M., Boldogkői Z. (2019). Multiple Long-Read Sequencing Survey of Herpes Simplex Virus Dynamic Transcriptome. Front. Genet..

[31] Prazsák I., Moldován N., Balázs Z., Tombácz D., Megyeri K., Szűcs A., Csabai Z., Boldogkői Z. (2018). Long-read sequencing uncovers a complex transcriptome topology in varicella zoster virus. BMC Genom..

[32] Balázs Z., Tombácz D., Szűcs A., Csabai Z., Megyeri K., Petrov A.N., Snyder M., Boldogkői Z. (2017). Long-Read Sequencing of Human Cytomegalovirus Transcriptome Reveals RNA Isoforms Carrying Distinct Coding Potentials. Sci. Rep..

[33] Balázs Z., Tombácz D., Szűcs A., Snyder M., Boldogkői Z. (2017). Long-read sequencing of the human cytomegalovirus transcriptome with the Pacific Biosciences RSII platform. Sci. Data.

[34] Moldován N., Tombácz D., Szűcs A., Csabai Z., Balázs Z., Kis E., Molnár J., Boldogkői Z. (2018). Third-generation Sequencing Reveals Extensive Polycistronism and Transcriptional Overlapping in a Baculovirus. Sci. Rep..

[35] Moldován N., Balázs Z., Tombácz D., Csabai Z., Szűcs A., Snyder M., Boldogkői Z. (2017). Multi-platform analysis reveals a complex transcriptome architecture of a circovirus. Virus Res..

[36] Moldován N., Szűcs A., Tombácz D., Balázs Z., Csabai Z., Snyder M., Boldogkői Z. (2018). Multi-platform next-generation se-quencing identifies novel RNA molecules and transcript isoforms in an endogenous retrovirus. FEMS Microbiol. Lett..

[37] Olasz F., Tombácz D., Torma G., Csabai Z., Moldován N., Dörmő Á., Prazsák I., Mészáros I., Magyar T., Tamás V. (2020). Short and Long-read Sequencing Survey of the Dynamic Transcriptomes of African Swine Fever Virus and the Host Cells. Front. Genet..

[38] Tombácz D., Prazsák I., Szűcs A., Dénes B., Snyder M., Boldogkői Z. (2018). Dynamic transcriptome profiling dataset of vaccinia virus obtained from long-read sequencing techniques. GigaScience.

[39] Tombácz D., Balázs Z., Csabai Z., Moldován N., Szűcs A., Sharon D., Snyder M., Boldogkői Z. (2017). Characterization of the Dynamic Transcriptome of a Herpesvirus with Long-read Single Molecule Real-Time Sequencing. Sci. Rep..

[40] Boldogkői Z., Moldován N., Balázs Z., Snyder M., Tombácz D. (2019). Long-Read Sequencing—A Powerful Tool in Viral Transcriptome Research. Trends Microbiol..

[41] Wick R.R., Judd L.M., Holt K.E. (2019). Performance of neural network basecalling tools for Oxford Nanopore sequencing. Genome Biol..

[42] Tombácz D., Prazsák I., Csabai Z., Moldován N., Dénes B., Snyder M., Boldogkői Z. (2020). Long-read assays shed new light on the transcriptome complexity of a viral pathogen. Sci. Rep..

[43] Balázs Z., Tombácz D., Csabai Z., Moldován N., Snyder M., Boldogkői Z. (2019). Template-switching artifacts resemble alternative polyadenylation. BMC Genom..

[44] Tombácz D., Csabai Z., Oláh P., Havelda Z., Sharon D., Snyder M., Boldogkői Z. (2015). Characterization of novel transcripts in pseudorabies virus. Viruses.

[45] Ardui S., Ameur A., Vermeesch J.R., Hestand M.S. (2018). Single molecule real-time (SMRT) sequencing comes of age: Applications and utilities for medical diagnostics. Nucleic Acids Res..

[46] Mortazavi A., Williams B.A., McCue K., Schaeffer L., Wold B. (2008). Mapping and quantifying mammalian transcriptomes by RNA-Seq. Nat. Methods.

[47] Djebali S., Davis C.A., Merkel A., Dobin A., Lassmann T., Mortazavi A., Tanzer A., Lagarde J., Lin W., Schlesinger F. (2012). Landscape of transcription in human cells. Nature.

[48] Steijger T., Abril J.F., Engström P.G., Kokocinski F., Hubbard T.J., Guigó R., Harrow J., Bertone P. (2013). Assessment of transcript reconstruction methods for RNA-seq. Nat. Methods.

[49] Rhoads A., Au K.F. (2015). PacBio Sequencing and Its Applications. Genom. Proteom. Bioinform..

[50] Boldogkői Z. (2012). Transcriptional interference networks coordinate the expression of functionally-related genes clustered in the same genomic loci. Front. Genet..

[51] Gilet J., Conte R., Torchet C., Benard L., Lafontaine I. (2019). Additional Layer of Regulation via Convergent Gene Orientation in Yeasts. Mol. Biol. Evol..

[52] Fire A., Xu S., Montgomery M.K., Kostas S.A., Driver S.E., Mello C.C. (1998). Potent and specific genetic interference by double-stranded RNA in Caenorhabditis elegans. Nature.

[53] Tombácz D., Sharon D., Szűcs A., Moldován N., Snyder M., Boldogkői Z. (2018). Transcriptome-wide survey of pseudorabies virus using next- and third-generation sequencing platforms. Sci. Data.

[54] Rosel J.L., Earl P.L., Weir J.P., Moss B. (1986). Conserved TAAATG sequence at the transcriptional and translational initiation sites of vaccinia virus late genes deduced by structural and functional analysis of the HindIII H genome fragment. J. Virol..

[55] Nolte H., MacVicar T.D., Tellkamp F., Krüger M. (2018). Instant Clue: A Software Suite for Interactive Data Visualization and Analysis. Sci. Rep..

