# Supplementary material for: Time-Course Transcriptome Profiling of a Poxvirus Using Long-Read Full-Length Assay
Source: Pathogens. 2021 Jul 21;10(8):919. doi: 10.3390/pathogens10080919 (PMC8398953; doi:10.3390/pathogens10080919)
Supplement: Supplementary file 1 [file pathogens-10-00919-s001.zip › suppl/Figure S2.pdf]

GENE	TRANSCRIPT	STRAND	LENGTH (bp)	Relative abundance of 3' UTR isoforms						
				1h	2h	3h	4h	6h	8h	12h
C11R	C11R	+	486	0	0.06667	0	0	0	0	0
	C11R-AT1	+	515	0.6	0.86667	0.6	0.5	0.71429	1	0.58333
	C11R-AT2	+	529	0.4	0.06667	0.4	0.5	0.14286	0	0.41667
	C11R-AT3	+	551	0	0	0	0	0.14286	0	0
C10L	C10L	-	1046	0.666667	0.2	1	0.8	0.33333	1	0.5
	C10L-AT1	-	1057	0.166667	0.6	0	0.2	0.33333	0	0.125
	C10L-AT2	-	1079	0	0	0	0	0	0	0.125
	C10L-AT3	-	1102	0.166667	0	0	0	0	0	0
	C10L-AT4	-	1115	0	0.2	0	0	0.33333	0	0.25
VACWR_15	VACVWR_15	-	304	0.5	0.15789	0.142857	0.2	0.08333	0.333333	0.23077
	VACVWR_15-AT1	-	318	0.5	0.15789	0.142857	0.26667	0.33333	0.166667	0.15385
	VACVWR_15-L	-	384	0	0.26316	0.142857	0.26667	0.16667	0	0.53846
	VACVWR_15-AT1-l	-	398	0	0.42105	0.571429	0.26667	0.41667	0.5	0.07692
C6L	C6L	-	546	0	1	1	1	0.5	1	0.66667
	C6L-AT1	-	559	0	0	0	0	0.25	0	0.33333
	C6L-C7L	-	1286	0	0	0	0	0.25	0	0
C5L	C5L	-	697	1	1	1	1	1	1	0.8
	C5L-C6L	-	1263	0	0	0	0	0	0	0.2
C4L	C4L	-	1041	0	0.5	0	0	0	0	0
	C4L-AT1	-	1156	0	0.5	0	0	0	0	0
N1L	N1L-C1L	-	1088	0	0	0	0	0	0.2	0
	N1L-C1L-l	-	1133	1	1	1	1	0.25	0.8	0
	N1L-C1L-AT1	-	1181	0	0	0	0	0.25	0	0
	N1L-C1L-l-AT1	-	1226	0	0	0	0	0.25	0	0
	N1L-C1L-C2L	-	2709	0	0	0	0	0.25	0	0
K1L	K1L	-	921	0.9375	0.25	0.833333	1	0.85714	0.8	1
	K1L-AT1	-	951	0.0625	0.375	0	0	0	0	0
	K1L-l	-	1192	0	0	0.166667	0	0.14286	0	0
	K1L-AT1-l	-	1222	0	0.25	0	0	0	0	0
	K1L-AT2-l	-	1244	0	0	0	0	0	0.2	0
	K1L-M2L	-	1743	0	0.125	0	0	0	0	0
K7R	K7R	+	493	0.8	0.84615	1	1	1	1	0
	K7R-AT	+	522	0.2	0.15385	0	0	0	0	0
F1L	F1L	-	855	0	0.0303	0.25	0	0	0.055556	0
	F1L-AT3	-	896	0.090909	0	0	0	0	0	0
F2L	F2L-F1L-AT1	-	1138	0	0.06061	0	0	0.07143	0	0
	F2L-F1L	-	1170	0.727273	0.78788	0.75	0.71429	0.64286	0.666667	1
	F2L-F1L-AT2	-	1188	0	0.06061	0	0	0	0.111111	0
	F2L-F1L-AT3	-	1211	0.181818	0.0303	0	0.14286	0.07143	0.111111	0
	F2L-F1L-AT4	-	1223	0	0.0303	0	0	0.14286	0.055556	0
	F2L-F1L-AT5	-	1254	0	0	0	0.14286	0.07143	0	0
F4L	F4L	-	986	0.8	0.82353	1	0.5	0.85714	0.888889	0.875
	F4L-AT1	-	1000	0.2	0.17647	0	0.5	0.14286	0.111111	0.125
F5L	F5L	-	1401	0	0	0	0	0	0	0.5
	F5L-F4L	-	1982	1	0	0	0	0	1	0.5
VACWR_43R	VACVWR_43	-	320	0.7	0.72727	0.875	0.91667	0.5	0.75	0.75
	VACVWR_43-AT2	-	333	0.3	0.24242	0.125	0.08333	0.5	0.25	0.25

	VACVWR_43-F6L	-	553	0	0.0303	0	0	0	0	0
F14L	F14L	-	280	0.5	0.77778	1	0.75	0	1	1
	F14L-AT2	-	313	0	0.11111	0	0.25	0	0	0
F15L	F15L	-	517	0	0.11111	0	0	0	0	0
	F15L-F14.5L-F14L	-	996	0.5	0	0	0	0	0	0
E3L	E3L-AT1	-	663	0	0	0	0.07692	0	0	0.125
	E3L-AT2	-	680	0	0	0.083333	0.07692	0.08333	0	0
	E3L	-	695	0.857143	0.82143	0.916667	0.69231	0.66667	0.875	0.875
	E3L-AT3	-	723	0	0.03571	0	0	0	0	0
	E3L-AT4	-	737	0	0	0	0.07692	0	0.125	0
	E3L-AT7	-	848	0	0	0	0	0.08333	0	0
E4L	E4L-E3L-AT1	-	1507	0	0.03571	0	0	0	0	0
	E4L-E3L	-	1522	0.142857	0.10714	0	0.07692	0.16667	0	0
O1.2L	O1.2L-AT2	-	429	0	0	0	0	0.33333	0	0
	O1.2L-AT1	-	451	0	0	0	0	0	0	1
	O1.2L	-	462	0	0.11111	0.25	0	0.33333	0	0
O1L	O1L-AT1	-	2046	0	0.22222	0	0	0	0.333333	0
	O1L	-	2057	1	0.66667	0.75	1	0.33333	0.666667	0
I3L	I3L	-	1031	1	1	1	0	0	0.5	0
	I3L-AT2	-	1044	0	0	0	0	0	0.5	0
L2R	L2R	+	289	0	1	0	0	1	0	1
	L2R-AT1	+	538	1	0	0	0	0	0	0
J2R	J2R	+	590	0.666667	0.55556	1	0.5	0.72727	1	0.6
	J2R-AT1	+	605	0.333333	0.11111	0	0.25	0.09091	0	0.4
	J2R-AT2	+	628	0	0.33333	0	0.25	0.09091	0	0
	J2R-AT3	+	708	0	0	0	0	0.09091	0	0
H5R	H5R	+	736	0.8	0.625	0.5	0.64	0.71429	0.772727	0.28571
	H5R-AT1	+	758	0.2	0.325	0.428571	0.32	0.19048	0.181818	0.57143
	H5R-AT3	+	899	0	0.025	0.071429	0.04	0.09524	0.045455	0.14286
	H5R-AT4	+	912	0	0.025	0	0	0	0	0
D7R	D7R	+	519	0	0.75	0	0	0	1	0
	D7R-AT	+	542	1	0.25	0	0	0	0	1
A12.2L-as-nc-A11R	A12.2L-as-nc-A11R	-	190	0	0	0	1	0	0	0
	A12.2L-as-nc-A11R-	-	333	0	0	0	0	1	0	0
A33R	A33R	+	616	0.7	0.82609	0.75	0.33333	0.6	1	0.86667
	A33R-AT1	+	651	0.3	0.17391	0.25	0.66667	0.2	0	0.13333
	A33R-AT2	+	705	0	0	0	0	0.2	0	0
A35R	A35R	+	570	0	0.85714	1	1	0.33333	1	0
	A35R-AT1	+	587	0	0.14286	0	0	0.66667	0	0
A37R	A37R-AT3	+	952	0	0	0.25	0	0	0	0.25
	A37R	+	976	0.583333	0.76	0.75	0.5	0.5	0.5	0.5
	A37R-AT1	+	1019	0.166667	0.2	0	0.25	0.25	0	0.25
	A37R-AT2	+	1034	0.083333	0	0	0	0	0	0
	A37R-AT6	+	1069	0.166667	0	0	0	0.125	0.5	0
	A37R-AT7	+	1100	0	0.04	0	0.125	0	0	0
	A37R-VACVWR_161	+	1125	0	0	0	0.125	0	0	0
	A37R-VACVWR_161	+	1184	0	0	0	0	0.125	0	0
A37.5R	A37.5R	+	401	0	0	0	0.1	0	0	0
	A37.5R-AT	+	525	0	0	0	0.1	0	0	0

VACWR_161	VACVWR_161-l-AT1	+	231	0	0.5	0.5	0	0	0	0
	VACVWR_161-l	+	338	0	0	0	0	0	0	0.66667
	VACVWR_161-l-AT3	+	367	0	0	0.5	0	0	0	0
	c-VACVWR_161-l-A[illegible]	+	1083	0	0.5	0	0	0	0	0.33333
A40R	A40R-AT1	+	535	0	0	0	0	0	0	0.33333
	A40R	+	552	0	1	0	1	1	1	0.66667
VACWR_169	VACVWR_169	+	295	1	0.92857	0.833333	1	1	1	1
	VACVWR_169-AT1	+	357	0	0.07143	0.166667	0	0	0	0
A46R	A46R	+	744	0.4	0	0	0	0	0	0
	A46R-AT1	+	771	0.6	1	1	0	1	1	1
A51R	A51R-AT2	+	1041	0	0.66667	0.5	0	0	0	0.5
	A51R	+	1057	1	0.33333	0.5	0.75	1	1	0
	A51R-AT5	+	1118	0	0	0	0.25	0	0	0.5
B2R	B2R	+	807	0	0	0	0	0	0.166667	0
	B2R-VACVWR_187-[illegible]	+	1635	0.166667	0	0	0	0	0	0
	B2R-VACVWR_187	+	1732	0.666667	1	0	1	0	0.666667	0
	B2R-VACVWR_187-[illegible]	+	1792	0.166667	0	0	0	0	0.166667	1
B8R	B8R	+	885	0.6	0.81395	1	0.93103	0.91667	0.785714	0.94286
	B8R-AT3	+	1089	0	0	0	0	0.08333	0	0.02857
B13R	B13R-AT1	+	1108	0	0.08333	0	0	0	0	0.5
	B13R	+	1151	1	0.91667	0.5	1	0	1	0.5
	B13R-AT2	+	1168	0	0	0.5	0	0	0	0
B19R	B19R	+	1117	1	1	1	1	1	0.961538	0.97143
	B19R-AT1	+	1168	0	0	0	0	0	0	0.02857
	B19R-AT2	+	1290	0	0	0	0	0	0.038462	0
C10L	C10L	+	1046	0.833333	0.92857	1	0.9	0.33333	1	0.5
	C10L-AT1	+	1057	0	0	0	0.1	0.33333	0	0.125
	C10L-AT2	+	1089	0	0	0	0	0	0	0.125
	C10L-AT3	+	1102	0.166667	0	0	0	0	0	0
	C10L-AT4	+	1115	0	0.07143	0	0	0.33333	0	0.25
B29R	B29R-AT1	+	826	0	0	0	0	0	0.142857	0
	B29R-AT3	+	970	0	0	0.25	0	0	0	0
	B29R	+	1005	1	1	0.75	1	1	0.857143	1
